# The moral significance of moral distress

**DOI:** 10.1007/s11019-026-10370-2

**Published:** 2026-06-22

**Authors:** Cullin Brown, Jan Helge Solbakk

**Affiliations:** 1 Centre for Medical Ethics, University of Oslo, Oslo, Norway; 2 Department of Philosophy, Binghamton University, Binghamton, NY USA

**Keywords:** Moral distress, Moral failure, Fitting emotions, Healthcare providers, Acceptance

## Abstract

Since Andrew Jameton introduced the term “moral distress” (MD) in 1984, the concept has attracted extensive attention in the healthcare literature. Much of this work emphasizes the costs of MD–for instance, psychological pain, work dissatisfaction, and burnout–while some highlights its potential benefits, including motivating self-improvement and institutional reform. We survey these assessments and argue that the bulk of them share a common limitation: they evaluate MD purely instrumentally, in terms of its painfulness and consequences, rather than its character as a moral experience. This instrumental framing, we contend, obscures MD’s core moral significance and invites misguided interventions. In particular, it neglects the question of when MD is a fitting response to one’s situation, and thus justified on intrinsic rather than instrumental grounds. We suggest that MD is fitting when one is genuinely constrained from meeting a moral requirement, and that fitting MD is significant in its own right.

## Introduction

In 1984, Andrew Jameton defined *moral distress* (MD) as the distress that “arises when one knows the right thing to do, but institutional constraints make it nearly impossible to pursue the right course of action” (p. 6). Since then, the concept has received extensive attention in the healthcare literature. Much of this work includes an evaluation or assessment of MD–generally as negative, given its costs (e.g., psychological pain, work dissatisfaction, and burnout), but sometimes as positive, given its potential benefits (e.g., catalyzing moral development and institutional reform). We survey these evaluations and argue that they share a common limitation: by assessing MD primarily in terms of its painfulness and effects, they displace its moral-experiential core. In other words, this instrumental approach overlooks MD’s significance *as a moral experience*.

More specifically, healthcare professionals (HCPs) experiencing MD construe themselves, through their thoughts and emotions, in a particular moral light–namely, as constrained from doing what is morally required of them. This self-construal may be accurate or inaccurate, fitting or unfitting, and MD may therefore be justified on intrinsic rather than instrumental grounds–that is, because it gets things *right*. Accordingly, we suggest that the primary (i.e., first and essential) sense in which MD is significant is moral-experiential rather than psychological or consequential: when it is fitting, it emotionally registers the moral reality of one’s situation. For this reason, moreover, interventions should not–except in extreme cases–aim to eliminate fitting MD. Instead, its fittingness should be acknowledged and affirmed, and, where further response is needed, we should work with rather than against it–for instance, by helping HCPs channel their MD into constructive action or, where this is not possible, by supporting them in accepting the reality of their (perhaps unavoidable) moral failure and bearing its burden.

Two preliminary notes: First, this paper began as a systematic review, aimed at categorizing the evaluations of MD that have appeared since Jameton’s 1984 account. We conducted an initial search in Scopus (a multidisciplinary database that covers nearly all of PubMed/Medline content) for “moral distress” and related terms, combined with terms for HCPs and their practices.^1^ As we pored over the results, however, we were struck by how instrumental these evaluations tended to be, and our interests shifted from developing a bottom-up taxonomy of evaluations of MD to holding these evaluations accountable to MD’s nature as a moral experience. In light of this shift, we adopted a looser, narrative methodology. We screened the abstracts of papers identified through our initial and subsequent searches for indications that the paper advanced an evaluation of MD, read the papers that appeared promising, and retained those that we judged to in fact advance such an evaluation.^2^ While many of the papers that we retained are empirical in orientation, they nonetheless make substantive evaluative claims about MD–typically in their introductions and conclusions, but more robustly than is usual for merely motivating one’s research. We then grouped the retained papers thematically, based on the kinds of assessments they advance and the assumptions about MD’s value and nature that underlie them (see Table 1).^3^ We maintain that our survey is representative–and we have preserved certain systematic features of our search in order to support this claim–but we do not claim that it is exhaustive, and we acknowledge that our groupings are more associative than analytic.

**Table 1:** Papers retained for review

Category	Number of papers	References
MD as an indicator of institutional moral failure	2	Jameton (1984); Jameton (2017);
MD as instrumentally negative	39	Abbasi, Ghafari, Shahriari, et al. (2019); Andersson, Fredholm, Nordin, et al. (2023); Atli Özbaş and Kovancı (2022); Beil-Hildebrand, Sari Kundt, Kutschar, et al. (2024); Beltrão, Beltrão, Bernardelli, et al. (2023); Bong (2019); Brandon, Ryan, Sloane, et al. (2014); Canciani et al. (2016); Carletto, Ariotti, Garelli, et al. (2022); Cavaliere, Daly, Dowling, et al. (2010); De Veer, Francke, Struijs, et al. (2013); Ferrell (2006); Getahun, Gurara, Bekele, et al. (2024); Ghahremani, Esmaelzadeh, Khosravani, et al. (2022); Gutierrez (2005); Hickey (2022); Hiler, Hickman, Reimer, et al. (2018); Imbulana et al. (2021); Janatolmakan, Dabiry, Rezaeian, et al. (2021); Kalani et al. (2023); Kiziltepe and Kurtgöz (2022); Ko, Tseng, Chin, et al. (2020); Lewis (2019); Lusignani, Giannì, Re, et al. (2017); McAndrew and Hardin (2020); Molazem, Tavakol, Sharif, et al., (2013); Palmer, McCullough, Wormwood, et al, (2023); Park, Thrul, Cooney, et al. (2024); Pereira et al. (2023); Poisson, Alderson, Caux, et al. (2014); Rabin, Kika, Lamb, et al. (2023); Rathert et al. (2016); Sarro, Haviland, Chow, et al. (2022); Tajalli, Rostamli, Dezvaree, et al. (2021); Tavakol, Molazem, Rakhshan, et al. (2023); Torjuul and Sorlie (2006); Wilkinson (1987); Wilson, Simmons, Harris, et al. (2022); Witton et al. (2023)
MD as instrumentally positive or mixed	8	Benner (1991); Corley (2002); Harding (1980); Laabs (2005); Lützén, Cronqvist, Magnusson, et al. (2003); Prentice, Gillam, Davis, et al. (2018); Varcoe, Pauly, Storch, et al. (2012); Wiegand and Funk (2012);
Moral resilience	11	Chen, Zhang, Zheng, et al. (2023); Metselaar and Molewijk (2023); Monteverde (2013); Rushton (1992); Rushton (2016); Rushton, Batcheller, and Schroeder (2015); Rushton et al. (2017); Rushton, Swoboda, and Reller (2021); Sala Defilippis et al. (2019); van Schaik, Pasman, Widdershoven, et al. (2024); Wros, Mathews, Beiers-Jones, et al. (2021)
Non-instrumental/moral significance	7	Fiester (2023); Johnstone and Hutchinson (2015); Lee and Kim (2020); Morley et al. (2020); Peter (2015); Russell (2012); Tessman (2020)
Total	67	

Second, in order to make the points that we wish to make, we need to adopt a working understanding of MD. In a companion paper, we argue that MD is best understood as “the anguished or panicked sense that one is constrained from doing what one takes to be morally required,” or, put differently, “the felt collision of a moral ‘*I must*’ with an ‘*I can’t*’” (Brown and Solbakk 2026). This account is motivated by the view that MD has a moral-experiential core that other accounts have largely overlooked, generally by treating it as a negatively valenced psychological state that happens to arise in a moral context. The present paper is similarly motivated, and we therefore adopt this understanding of MD here. That said, our central claims require only the more modest assumption that HCPs experiencing MD can be meaningfully understood as construing themselves, through their thoughts and emotions, in a particular moral light, such that MD can be a fitting or unfitting response to their situation. Those who are not persuaded by our specific understanding of MD are thus welcome to treat it as a placeholder or substitute their preferred alternative, so long as it preserves this core acknowledgement.

## Moral distress as an indicator of institutional moral failure

We begin with Jameton, who not only gave MD its canonical formulation but set the terms for its evaluation by characterizing it as a *problem*. On his view, MD is a symptom of moral failure at the institutional level: healthcare organizations are morally deficient insofar as they constrain HCPs–through their authority structures, policies, cost-containment measures, and the like–from pursuing the right course of action (Jameton 1984, pp. 7–15). Thus, in contrast to later theorists who, as we will see, tend to regard MD as problematic primarily because of its detrimental effects on HCPs’ well-being and the consequences that follow, Jameton understands MD as problematic because it reflects a *moral* problem. We take this to be an important virtue of Jameton’s account, as it locates MD’s primary significance within the moral domain, rather than the psychological or occupational domains. In doing so, moreover, it rightly draws attention to the moral significance of the conditions under which HCPs practice and to the ways in which those conditions can constrain or inhibit moral agency.

At the same time, however, Jameton’s evaluation is limited in two key respects. First, by tying his evaluation of MD to institutional deficiencies, Jameton risks casting off experiences of MD that are not caused by institutional constraints as morally insignificant. In our companion paper, we identify three broad types of constraints: institutional constraints, as emphasized by Jameton; internal constraints, as emphasized by later theorists (e.g., lack of medical competence and lack of moral courage; see, for example, Wilkinson, 1989; Epstein and Hamric 2009); and human constraints, which arise from our condition as finite agents navigating a messy world rather than from any flaw or shortcoming (Brown and Solbakk 2026). An example of MD arising from a human constraint is an HCP caught between a patient’s wish to forgo life-sustaining treatment and family members’ pleas to continue it. Even if one of these considerations is overriding, the other may still be experienced as binding, and thus present itself as a constraint as soon as the HCP turns their focus to acting on the other. Crucially, the constraint in this case does not stem from any flaw or shortcoming on the part of the agent or the institution, but from our principled inability to act in a way that preserves all of the values at stake (Brown and Solbakk 2026).^4^  

If the moral significance of MD were exhausted by its connection to institutional moral failure, then cases arising from internal and human constraints would be morally irrelevant or at least derivative. But this is implausible. MD that arises, for example, from a tragic conflict between equally weighty obligations, or from the recognition that one lacks the knowledge needed to do what one takes to be morally required, is surely morally significant. Thus, although Jameton’s evaluation highlights one way in which certain experiences of MD are morally significant–namely, as indicators of institutional moral failure–it does not speak to the moral significance of the experience of MD itself.

A more general concern, here, is that Jameton’s evaluative framework implicitly treats MD as morally significant only insofar as it functions as an indicator that something ought to be fixed. This orientation fits naturally with his emphasis on institutional constraints, since these constraints can, at least in principle, be remedied. However, once we broaden our focus to include human constraints, it becomes clear that not all instances of MD can be prevented (Brown and Solbakk 2026). But again, it is implausible that MD loses its moral significance when there is no fix, since one’s experience of MD may still accurately register the moral gravity or even the tragic nature of one’s situation. Thus, evaluations that focus exclusively on MD as a prompt for intervention overlook an important dimension of its significance.

A second limitation concerns how the status of an HCP’s moral convictions bears on the moral significance of their MD. Jameton assumes that the moral convictions that animate HCPs’ experiences of MD are correct (MD “arises when one *knows* the right thing to do” [1984, p. 6; emphasis added]). Thus, he does not address the question of how MD’s moral significance depends on the correctness–or, if one prefers a less exacting term, the tenability–of the moral convictions that underpin it. Johnstone and Hutchinson (2015) draw attention to this point with the following observation:

[W]here the rightness of the nurses’ ethical judgments is assumed rather than shown, there will remain an unacceptable risk of nurses promulgating ‘moral imposition’ whereby their own personal views are imposed onto others in ways that are not only unwelcome but can result in otherwise preventable harm to people’s significant moral interests. (p. 12)

This is a legitimate concern, especially when the point is expanded to include physicians and other HCPs. Moreover, it reflects a deeper theoretical problem: Jameton’s definition “commits the fallacy of ‘begging the question,’ that is, it ‘takes for granted… precisely what is in dispute,’ in this instance: the rightness of nurses’ moral judgments” (Johnstone and Hutchinson 2015, p. 12; citing Flew and Priest 2002, p. 43).^5^ In other words, being prevented from acting in accordance with one’s moral convictions is one thing, but the correctness of those convictions is another. By building the correctness of HCPs’ moral convictions into his definition and evaluation of MD, however, Jameton obscures this distinction and thereby sidelines questions about how it bears on the significance of one’s MD.

Here, too, there is a more general issue. Even if the moral conviction that underpins an HCP’s experience of MD is correct, there remains the question of whether the constraint they experience is real. This matters because one’s experience of a constraint may misrepresent what is in fact possible or exaggerate the magnitude of the barrier. For instance, perceived powerlessness may reflect a misperception rather than genuine powerlessness, or the professional risks of voicing disagreement may be overestimated (Johnstone and Hutchinson 2015, pp. 9–10). Surely both of these considerations–the correctness of one’s moral convictions and the reality of the constraint one experiences–bear on the significance of one’s MD. This is no accident, as these considerations are central to whether MD is a fitting response to one’s situation, a point we develop further below. The point for now is that fully accounting for the significance of MD requires assessing both the normative status of the relevant moral conviction and the reality of the experienced constraint. Yet theorists in the healthcare literature on MD have been largely unwilling to engage with the moral and metaethical questions that such assessments demand. These questions, as will become clear, are often bypassed or bracketed, perhaps because engaging with them is taken to be unscientific. But such engagement is indispensable when it comes to MD, lest one lose sight of its moral core.

### An instrumental turn

While Jameton frames MD as a moral problem rooted in institutional failure, much of the subsequent literature evaluates MD in a markedly different way. These accounts assess MD primarily in terms of its affective qualities–its unpleasant or aversive felt character–and its downstream effects. Wilkinson (1987) occupies an important transitional role in this shift. She characterizes MD in explicitly psychological terms, defining it as the “psychological disequilibrium and negative feeling state” that arise when an agent is prevented from acting in accordance with their moral judgments (1987, p. 16). At the same time, Wilkinson emphasizes that MD is not merely a matter of painful feelings but includes “situational, cognitive, and action dimensions as well as the feelings dimension” (1987, p. 24). Nevertheless, her emphasis on MD’s psychological features, as well as their effects on HCPs and patient care, marks a shift in focus. Later theorists tend to foreground MD’s affective qualities rather than its nature as a moral experience, emphasizing its painfulness and association with adverse outcomes.

Building on this shift, much of the subsequent literature develops this psychological framing into a more fully instrumental evaluation of MD. While much of this work is empirical in orientation, its findings are often taken to support a negative evaluation of MD grounded in its adverse effects, and thus to motivate efforts to mitigate or prevent it. At the personal level, MD has been associated with enduring anger, crying, guilt, malaise, pain, and sleep disorders (Ferrell 2006; Gutierrez 2005; Schaefer et al. 2016). These effects can contribute to burnout, compassion fatigue, and diminished mental health, and they may also lead to reduced job satisfaction and the decision to leave the profession (Canciani et al. 2016; Carletto et al. 2022; Cavaliere et al. 2010; Gutierrez 2005; Prentice et al. 2018). Moreover, because healthcare systems depend on the well-being and retention of their workforce, these individual-level consequences have broader institutional implications, including high staff turnover and diminished quality of care, such as reduced patient engagement and less personalized treatment (Carletto et al. 2022; Cavaliere et al. 2010; Gutierrez 2005; Prentice et al. 2018).

Despite the overwhelming emphasis on the harmful effects of MD, some theorists have highlighted its potential benefits, suggesting that it may play a constructive role in moral and professional development. For instance, Corley (2002) draws on earlier work to highlight what she terms the “benefits of moral distress,” including its capacity to prompt reflection on good patient care, reinforce professional commitments, and cultivate more skillful and compassionate practice. Similarly, Lützén et al. (2003) suggest that moral stress–their term for the broader phenomenon of which MD comprises the negative aspects–can function as an “energizing factor,” motivating agents to pursue professional goals and respond more effectively to morally challenging situations.

We do not doubt that this is *sometimes* the case. However, these more positive approaches are limited in a way that parallels the limitations of Jameton’s approach: by tying MD’s significance to its ability to catalyze improvement–whether in one’s own practice or in the surrounding institutional context–they risk casting off experiences of MD that do not point to any such improvement as morally insignificant. Lützén et al.’s distinction between moral stress and MD is especially noteworthy in this regard. On their view, moral stress–but not MD–involves *awareness* of the moral values at stake and of the constraints that make it difficult or impossible to uphold those values, and it is this awareness that is supposed to underwrite the more positive features of moral stress (2003, p. 314). However, when MD arises from human constraints, there is no corresponding avenue for improvement; increased moral awareness does not, in these cases, better equip one to uphold one’s moral values and may instead intensify one’s distress by bringing into sharper focus the absence of any clear resolution. Thus, while we agree that moral awareness is important, we worry that attempts to recast MD in more positive terms present an unduly optimistic picture.^6^

Even with this shift in evaluative valence, these positive accounts retain the same instrumental structure as their negative counterparts: MD is treated as good insofar as it produces desirable effects and bad insofar as it produces undesirable effects. In both cases, then, MD’s significance is understood in terms of what it does rather than what it is. This evaluative orientation contrasts not only with Jameton’s outward focus on institutional moral failure, but also with a different kind of inward focus–one concerned with MD’s significance as a moral experience.

## Moral resilience

A further development in the literature is the emergence of work on *moral resilience*, which builds on more positive evaluations of MD but shifts the focus from MD itself to the traits that might enable HCPs to respond to it in a healthy or constructive way. Rushton (2016), who helped introduce the concept of moral resilience in the context of MD, characterizes it as a “new paradigm” for thinking about MD–one that “focuses on cultivating moral resilience rather than on the despair and helplessness many clinicians experience” (p. 112). Drawing on broader understandings of resilience as “the ability to recover or healthfully adapt to challenges, stress, adversity, or trauma,” she defines moral resilience as “the capacity of an individual to sustain or restore their integrity in response to moral complexity, confusion, distress, or setbacks” (2016, p. 112).

We want to make two points about this proposal. The first is that efforts to specify how HCPs should respond to MD are no substitute for–and must instead rely on–a well-developed understanding of MD and its significance. How a morally resilient person would navigate an experience of MD depends, in part, on whether it is a fitting or unfitting response to their situation, as well as on whether the constraints that give rise to it might be remedied. To illustrate, consider the difference between fitting grief and a phobic fear. Grief construes one’s situation as involving a serious loss and, when fitting, emotionally registers the reality of that loss. For this reason, responding well to fitting grief plausibly involves accepting and carrying on with it–rather than trying to diminish it–as well as honoring who or what has been lost. Moreover, if the loss reflects one’s own shortcomings (for instance, a cherished relationship that was lost because of one’s character flaws), such a response may also involve self-reflection and change; if it could not have been prevented, such responses are not called for. By contrast, because phobic fears *mis*construe one’s situation as threatening, responding well to such a fear plausibly involves working to diminish or overcome it.

In much the same way, how a morally resilient person would respond to MD depends on whether their distress is fitting and, if so, whether it points to a fix or improvement that one might pursue. These are questions about the nature and significance of MD proper. Thus, while we do not object to efforts to develop a better understanding of moral resilience in the context of MD, we caution against treating such work as a substitute for a well-developed account of MD and its significance.

The second point is that Rushton’s account of moral resilience is problematically narrow. Her account centers on preserving or restoring one’s integrity (2016, p. 112), which is typically understood as coherence between one’s moral convictions and actions. Preserving one’s integrity is an appropriate focus when the (would-be) constraint can be addressed through self-work or institutional reform–for instance, when an HCP recognizes that their lack of moral courage threatens their integrity and can mitigate this threat by cultivating such courage. However, this focus misses the mark in cases of human constraint, since such constraints cannot be remedied. Consider, for example, an HCP who is responsible for treating a severely impaired newborn with poor prognosis. The clinician may feel morally required to do what is best for the newborn but unsure what that is, given their sense–correct, in our view–that notions such as benefits and harms do not bear determinately on this kind of case. At the same time, they might feel constrained from committing to any course of action–from aggressive treatment to palliative care–since each imposes an intolerable risk on the newborn (Brown and Solbakk 2026). In such cases, every available action stands in tension with the HCP’s moral convictions, and acting at all therefore involves compromising something that deeply matters; indeed, it would be troubling if the clinician did not experience some degree of moral fragmentation in these circumstances. Human constraints therefore highlight how being an HCP–and a finite moral agent in a messy world more generally–inevitably involves risking one’s moral integrity.

Similarly, restoring one’s integrity is an appropriate focus when moral repair is possible–for instance, when a patient survives and one can make amends. But it again misses the mark when repair is foreclosed–for instance, when a patient has died or been irreversibly harmed. In such cases, there may be no way to restore one’s integrity, at least not in the sense of wiping the slate clean. Rushton’s integrity-centric understanding of moral resilience is therefore inadequate for beings like us, or beings who are not in complete control of their moral wholeness.

A more promising approach is suggested by Metselaar and Molewijk (2023), who argue that moral case deliberation (MCD) can help foster moral resilience. MCD involves structured reflection on morally troubling cases, with an emphasis on participants’ experiences, emotions, and judgments rather than abstract theory (Metselaar and Molewijk 2023, p. 732). Crucially, some forms of MCD, such as the “Dilemma Method,” explicitly acknowledge that not all moral challenges admit of resolution. This method “supports the recognition that, sometimes, serious harm or suffering cannot be avoided, whichever choice is made” (Metselaar and Molewijk 2023, p. 741). It therefore resists “the idea that a ‘good professional’ should always be able to overcome moral adversity” (p. 734).

This aspect of MCD gestures toward a broader understanding of moral resilience.^7^ By explicitly acknowledging the tragic dimensions of certain moral situations, the Dilemma Method recognizes that there is not always an agential or institutional fix in the background of MD and, with this, that one’s integrity cannot always be preserved or restored. If moral resilience is to offer guidance in such cases, it cannot be understood solely in terms of maintaining or recovering moral wholeness. Rather, it must also involve the capacity to acknowledge and accept that one’s integrity can be unavoidably compromised, and to carry on with dignity regardless of one’s fragmented state. Such an account of moral resilience would require further development, but the central point is that an adequate conception must be indexed to the more thoroughly tragic features of the human condition.

## Moral distress as a fitting emotional response

The preceding discussion suggests that existing evaluations of MD do not fully capture what is at stake. By focusing on MD’s function, painfulness, effects, and management, they overlook its non-instrumental significance as a moral experience. A minority of accounts do better in this regard, but even they tend to downplay this experiential element. For instance, Fiester (2023) argues that some constraints on HCPs’ moral agency are justified, and that helping HCPs recognize this may reduce MD arising from such constraints–namely, by enabling HCPs to temper their distress in light of the broader moral picture. Yet the focus remains on reducing MD, such that the experience itself is decentered.

We have found only one publication that centers the experience of MD in accounting for its significance (Tessman 2020), which we discuss after laying the relevant groundwork. We then propose a modified version of Tessman’s view, which we offer as a supplement–although a central one–to other evaluations of MD, rather than as a wholesale objection to them.

An account of when an emotional response is fitting is necessarily conceptual rather than empirical. Still, such an account may be empirically informed. Our approach draws on sentimentalist and response-dependent views, which assign emotional responses a principled role in the anatomy of morality and which theorize, in various ways, from those responses to moral conclusions (see, for example, Hume, 1739/2000, 3.1.1; Strawson, 1962; and D’Arms and Jacobson 2023). It also draws on a philosophical tradition encompassing the literatures on moral residue, tragic conflicts, and dirty hands (see, for example, Tessman 2014; Walzer 1973; and Williams 1973 & 1981). These literatures emphasize that an agent may appropriately experience regret, anguish, or distress even when they act impeccably. Indeed, Williams argues that attending to “the facts of regret and related considerations” leads to the conclusion that “moral conflicts are neither systematically avoidable, nor all soluble without remainder” (1973, p. 179). The broader point is that these emotional responses should not be dismissed because of how they unsettle more conventional moral theorizing, but instead merit attention because of their potential to capture otherwise overlooked contours of our moral sensibilities.

We accept a refined version of D’Arms and Jacobson’s (2000) understanding of fittingness, according to which an emotion is fitting when “it accurately presents its object as having certain evaluative features” (p. 65). Thus, amusement, for example, is fitting when its object–what it is a response to, say, a joke–is genuinely funny. We introduce two refinements to this account. First, whereas it applies to the emotions proper–what are sometimes called the “basic” or “natural” emotions (see, for example, Izard 2007)–we broaden its scope to include emotional responses more generally. As we intend the term, emotional responses include the basic emotions as well as what Izard (2007) calls “emotion schemas,” or responses characterized by “the dynamic interaction of emotion and cognition” (p. 265; see also D’Arms and Jacobson 2003). Although MD is not a basic emotion, it qualifies as an emotional response in this broader sense because it would not be the experience that it is without its emotional components; someone who lacked acquaintance with these components could not fully grasp the experience.^8^

Second, emotional responses can construe their objects as having different kinds of normative features. Some construe their objects in an evaluative light–as good or bad in particular ways, such as delightful or disgusting. Others, by contrast, construe their objects in a deontic light–as involving requirements or prohibitions. Prinz (2009) refers to the emotional responses in this latter category as “oughtitudes” or “prescriptive sentiments.” Our understanding of MD includes such oughtitudes, most centrally the felt sense that something is morally required–the moral “I *must*.” The corresponding “I *can’t*” that figures in experiences of MD can be deontic as well, namely when it reflects a sense of moral prohibition (as in moral dilemmas), rather than descriptive impossibility (as in cases of physical constraint). We therefore broaden D’Arms and Jacobson’s account to include both evaluative and deontic features, which we group under the umbrella of “normative features.”

It will be helpful, finally, to distinguish between considerations that bear on whether an emotional response is fitting and considerations that bear on the response’s instrumental value or disvalue. We call these “reasons of fit” and “extrinsic reasons,” respectively.^9^ Thus, the fact that one’s boss told a joke may be an extrinsic reason for amusement–say, because it is prudent to be liked by one’s boss–but it is not a reason of fit because it does not speak to the question of whether the joke is genuinely funny. Conversely, that the joke used multiple meanings of the same word to establish and then subvert expectations is a reason of fit for amusement.

Extrinsic reasons can be quite strong, but reasons of fit have an important claim to primacy. Adapting an example from Srinivasan (2018, p. 128), suppose that Ollie tells an unflattering story about Amy in public, and that Amy becomes angry with him in response. If Ollie tells her to calm down because her anger is only going to make things worse, he thereby gives Amy an additional reason to be angry, namely that Ollie is not taking her seriously. More specifically, Ollie treats Amy’s anger as something that is merely to-be-managed–as akin, for example, to a headache (Hieronymi, 2019)–and therefore fails to engage with it as an expression of Amy’s normative perspective, her take on Ollie’s behavior as, say, a betrayal. Thus, by engaging with Amy’s anger only as something that might make things better or worse, Ollie bypasses its significance to her as a normative agent.

Crucially, this does not mean that Ollie must agree with Amy’s perspective; the problem, rather, is that he fails to engage with it *as a perspective* at all. Ollie might instead have argued that Amy’s anger is misplaced because sharing the story was an instance of gentle teasing rather than a betrayal, and there are, of course, more and less sensitive ways to voice such disagreement. But Ollie’s failure is more basic–and more condescending: he does not even recognize Amy’s perspective as worthy of engagement. Moreover, engaging with Amy’s anger as an expression of her normative perspective does not require Ollie to presuppose universally valid standards for what counts as gentle teasing versus a betrayal. It only requires him to recognize *some* standpoint as a source of reasons of fit, whether his own standpoint or the norms that help characterize the kind of relationship that he and Amy have with each other. In this way, the priority of reasons of fit is metaethically neutral: it rests only on the kind of taking one another seriously that characterizes ordinary interpersonal relationships (Strawson, 1962).

We take much of the literature on MD’s significance to mirror Ollie’s managerial approach to Amy’s anger: it gives scant–if any–attention to reasons of fit and instead emphasizes extrinsic reasons, especially those related to MD’s effects. In doing so, it sidelines MD’s significance as an expression of HCPs’ normative agency. Many of the instrumental considerations emphasized in this literature are indeed weighty; still, prioritizing them is a serious shortcoming, akin to focusing on a belief’s utility instead of its truth.^10^

Tessman (2020), as we have said, is an important exception. Central to her account of when MD is fitting is the category of the *unthinkable*, or actions that it would be wrong to even consider performing (p. 174), such as sacrificing one’s child. This category plays an important role both in loving relationships and in dependency relationships, where it matters–given the vulnerabilities involved–that certain forms of treatment are not even entertained. Because unthinkable actions are excluded from the realm of permissible deliberation, however, they are largely insulated from the processes through which we typically negotiate away one moral requirement when it conflicts with another. Thus, requirements that it would be unthinkable to violate can remain binding even when one is constrained from meeting them. This does not mean that it is impossible to do or think the unthinkable–indeed, the best option is sometimes unthinkable–but that doing so will always count as moral failure for which one bears responsibility, regardless of the constraints one faces (pp. 175-6).

On Tessman’s view, then, HCPs experiencing MD construe themselves as responsible for a moral failure, even though avoiding it would have been impossible or unreasonably demanding; this is the normative perspective that experiences of MD express. Moreover, MD is fitting only in cases involving the unthinkable, since only then does the (near) impossibility of avoidance leave intact the status of the action as a moral failure for which one bears responsibility. Nonetheless, encounters with the unthinkable are commonplace in healthcare, as HCPs are routinely “in situations in which their patients’ lives, their dignity, and their level of suffering depend on the practitioners’ actions,” and these “vulnerabilities give rise to their need to be treated with the intense quality of regard that the notion of unthinkability captures” (Tessman 2020, p. 175). HCPs’ experiences of MD are therefore often fitting.

As Tessman observes, finally, this has important implications for future research:

Beyond investigating how to reduce the *causes* of moral distress, the aim of future research should not be to reduce fitting moral distress itself. Instead, what is needed is exploration into methods to help medical professionals strike the right balance in their experiences of moral distress: the right balance will be one that avoids the unfairness of self-blame but that validates the fitting attitude, the attitude that accurately reflects the [normative] features of the action that was performed. (2020, p. 176)

We are broadly sympathetic to Tessman’s account, both substantively and methodologically–that is, in virtue of how it unpacks MD as an experience, articulates its significance as an experience, and prioritizes this significance in making recommendations for future research. We conclude this section by exploring one avenue for elaboration, although it is worth noting that disagreement at this level would still move the discourse in the direction we have been urging.

In a later paper, Jameton (1993) differentiates between *initial* and *reactive* forms of MD: “Initial distress involves the feelings of frustration, anger, and anxiety people experience when faced with institutional obstacles and conflict with others about values. Reactive distress is the distress that people feel when they do not act upon their initial distress” (p. 544). Tessman (2020) acknowledges that her account focuses on reactive MD (p. 167, n. 5), or what is sometimes called “moral residue” (see, for example, Epstein and Hamric 2009; Webster and Baylis 2000). However, she seems to assume that initial MD is not different in any theoretically significant way–that it is simply a prospective response that anticipates reactive MD.

Morley et al.’s (2020) findings on when initial MD translates into reactive MD call this assumption into question. Morley et al. suggest that Tessman’s (2017) distinction between moral conflicts and moral dilemmas helps explain when and why this translation occurs. On Tessman’s account, “a moral dilemma is a moral conflict in which neither of the two things that you’re morally required to do stops being required just because of the conflict” (pp. 27 − 8). This distinction is underwritten by that between ordinary moral requirements and the unthinkable, since a requirement remains binding in the presence of a constraint only when violating it would be unthinkable. Drawing on phenomenological interviews with critical care nurses, Morley et al. suggest that initial MD gives rise to reactive MD only when the experience is one of a moral dilemma and thus involves an encounter with the unthinkable:

When participants described moral conflicts, they described [initial MD], but they did not describe the associated residue because–applying Tessman’s definition to their accounts–the moral requirement in a moral conflict is negotiable: one moral requirement could be successfully negotiated for the other. Conversely, the moral requirement in a dilemma is non-negotiable, so although the moral agent may choose (for the purpose of action guidance) to fulfil one moral requirement instead of the other, this does not mean that it ceases to be a requirement. The residual guilt and regret [that constitute reactive MD] signals the continued pull of the non-negotiable moral requirement. (2020, p. 1309)

There are two main ways to interpret this result. One is that the initial MD arising in ordinary moral conflicts is unfitting, although psychologically intelligible given how such conflicts can resemble moral dilemmas (much as a harmless snake may elicit fear because of how it resembles a venomous one). The other is that initial MD is fitting across a broader range of cases than reactive MD: whereas reactive MD is fitting only in response to moral dilemmas/encounters with the unthinkable, initial MD may be a fitting response to moral conflicts/situations in which one is constrained from meeting a (would-be) moral requirement more generally.

Our initial sympathies lie with the second interpretation. Because even ordinary moral requirements do not typically come prepackaged in conditional form–as required *unless*…–to negotiate them away is to *do* something, and initial MD emotionally registers the moral struggle of (trying to) do this. That is, negotiable moral requirements are still moral requirements, and a felt “I *must*” is therefore a fitting response to them. Thus, when one is constrained from meeting such a requirement, the anguished or panicked sense that one cannot do what one must is fitting. The requirement’s negotiability gives one a way out of this predicament: one may permissibly release oneself from the requirement’s grip. Yet part of experiencing something as required, we suggest, is encountering at least some resistance upon trying to break free of its hold, even if one ultimately succeeds. Thus, initial MD that does not translate into reactive MD may accurately reflect the struggle involved in negotiating away a moral requirement. We therefore suggest that initial MD is fitting when one is constrained from meeting even an ordinary moral requirement, whereas reactive MD is fitting only when one has, in the face of a constraint, done something unthinkable. When MD is fitting, then, its primary significance is that it registers precisely this.

## Conclusion

We have argued that prevailing evaluations of MD are limited by their predominantly instrumental orientation, reflected both in their focus on MD’s effects and in their haste to reduce, channel, or otherwise manage it. The flipside of this orientation is a comparative neglect of MD’s significance *as a moral experience*. In an effort to recalibrate this state of affairs, we have emphasized how MD, as an emotional response, expresses a distinct normative perspective on one’s situation. Building on Tessman’s account of when MD is fitting, moreover, we have explored some of the nuances of when this perspective might get things *right*. We hope that the moral and metaethical questions that bear on this issue will receive greater attention in the healthcare literature on MD; to neglect them is to treat MD as a special kind of headache. This is especially important when it comes to developing interventions that respond to MD as a potentially fitting moral response, lest those whose MD is fitting, and for whom there is no positive outlet–those who are simply contending with the more tragic aspects of the human condition–be left empty-handed.

We conclude with some wisdom from Greek tragedy that may speak to this situation. In Sophocles’ second play about Oedipus, *Oedipus at Colonus*, the abdicated, old, and frail king makes his confession:

It’s little I ask, and get still less, but quite enough for me. Acceptance--that is the great lesson suffering teaches, suffering and the long years, my close companions. (1982, lines 1–7)

The expression πάθει µάθος (*pathei mathos*) originates from the play *Agamemnon* by Sophocles’ older colleague, Aischylos, and means “learning through suffering” or “living with and through suffering.” We take Oedipus to express precisely this idea when he acknowledges acceptance as “the great lesson suffering teaches.”

There is no shortage of things that might be fixed. But even if everything that might be fixed were fixed, there would still be the human condition to contend with, and one of its defining features is moral vulnerability. We would do well, accordingly, to accept this vulnerability–especially our vulnerability to being constrained from doing what we morally must–so that we might, like Oedipus, learn to bear the weight of moral suffering with clarity and dignity.^11^  

## Footnotes

1 Our key words included ‘moral distress’, ‘moral residue’, ‘moral failure/error’, ‘moral injury’, ‘culpable’, ‘culpability’, and ‘fallibility.’ Thank you to university librarian Marie Susanna Isachsen at UiO for assistance with this search.

2 We repeated our initial search on multiple dates, with the final search occurring on June 4, 2024. In total, our searches identified 2482 publications. 545 publications were removed either because they did not have abstracts (485 publications) or because they were published before Jameton’s 1984 work (60 publications). The remaining abstracts (1937 abstracts) were screened to determine whether the corresponding paper advanced an evaluation of MD. 67 publications were retained on these grounds.

3 Many of the papers we retained exhibit features of more than one category. We classify them according to their primary evaluative orientation. Most prominently, while many of these papers acknowledge that MD arises in morally salient contexts, this acknowledgement rarely plays a substantive role in their assessment of MD’s significance.

4 Although situations involving moral dilemmas (such as the case at hand) and moral uncertainty have traditionally been distinguished from MD, we argue that they can give rise to the same structure of experience–namely, the simultaneous experience of a moral “I *must*” and an “I *can’t*” (Brown and Solbakk 2026).

5 On these and other grounds, Johnstone and Hutchinson suggest “that the notion of moral distress ought to be abandoned” (2015, p. 5). We are more optimistic. In the present case, the corrective is for theorists to acknowledge that the moral convictions underlying HCPs’ experiences of MD are not invariably correct, and to engage directly with the substantive moral questions that this acknowledgement brings into view.

6 We take the same core point to apply to the updated distinction between moral stress and MD developed in Lützen and Kvist (2012).

7 Metselaar and Molewijk (2023) draw on Rushton’s integrity-centric account of moral resilience in motivating MCD. However, their discussion–especially of the Dilemma method–suggests a broader understanding of moral resilience, insofar as it acknowledges cases in which neither the preservation nor restoration of integrity is possible.

8 This is not to suggest that MD is invariably composed of the same emotional elements. Rather, there may be a range of affective variations that qualify as MD in virtue of their shared experiential core; those familiar with the phenomenon will generally be able to recognize these variations as instances of the same kind of experience.

9 Reasons of fit are often referred to as the “right kind of reasons,” and extrinsic reasons are often referred to as the “wrong kind of reasons” (Rabinowicz and Rønnow-Rasmussen 2004). This terminology can be confusing, however, since the distinction between good and bad reasons cuts across the distinction between the right and wrong kind of reasons. Thus, there can be bad reasons of the right kind–considerations that are taken to support the fittingness of an emotional response but that do not in fact support its fittingness–as well as good reasons of the wrong kind–considerations that in fact help establish how one should feel, all things considered, but not because they bear on the fittingness of that response. These odd-sounding phrases are artefacts of the context in which this distinction arose, namely the efforts of sentimentalists to isolate the reasons that support conclusions about which normative features an object has from the reasons that do not.

10 The force of this analogy depends, in part, on how objectively correct or incorrect one thinks our moral convictions can be, but it retains some force even on more subjectivist or otherwise relativist views: just as engaging with a belief as a claim about what is true–rather than explaining it away, say, as a product of the one’s upbringing–is central to taking the person who believes it seriously as an epistemic agent, engaging with an emotional response as a perspective on a situation’s normative features is central to taking the person experiencing it seriously as a normative agent.

11 We would like to thank Nikoletta Alexandri, Frank Hakemulder, Bert Molewijk, Lisa Tessman, and two anonymous reviewers for helpful discussions and feedback on earlier drafts of this paper.
